# Supplementary material for: Structure of Photosystem I Supercomplex Isolated from a Chlamydomonas reinhardtii Cytochrome b6f Temperature-Sensitive Mutant
Source: Biomolecules. 2023 Mar 15;13(3):537. doi: 10.3390/biom13030537 (PMC10046768; doi:10.3390/biom13030537)

Supplementary Materials

Strains, media and growth

The Chlamydomonas reinhardtii (C. reinhardtii) wild type (WT) strain 1A+ was supplied by Prof. Jean-David Rochaix.

The standard conditions best fitting the growth of the WT cultures were on TAP+ agar plates (Tris Acetate, Phosphate medium), at the temperature of 25°C under continuous illumination (70- 150 µE/m2/s). The liquid cultures were grown at 25°C on a shaker at a speed of 120 rpm (so the cells would not settle) under continuous illumination (20- 45 µE/m2/s). If not stated otherwise, cell growth was performed under these conditions. Alternatively, when stated that the cells were grown under non-permissive temperature conditions, the temperature used was of 37°C. The media was prepared as described elsewhere (Chlamy.org). TAP+ which is supplied with acetate was used for mixotrophic growth. TAP- lacks any organic carbon source and was used for photoautotrophic growth. All cell transfers and liquid culture dilutions were performed under sterile conditions given by a biological hood. Before being introduced to the living cells, all medium types and culture containers were sterilized for 40 minutes at 121°C by an autoclave.

UV mutagenesis

To induce random mutagenesis, we plated a 100 µl of fresh liquid cultures of the WT *C. reinhardtii* at cell density of 0.002 OD at 730nm and exposed it to UV light at 1 $\mu E/m^2/s$ for 2:45 - 3:45 minutes. A 80%-90% rate of cell death was recorded. The number of cells plated before exposure was aimed to reach 50-100 colonies per plate. Sets of UV treated plates that did not show the expected death rates in comparison to the corresponding control plates were not used. Plates with crowded colonies distribution (acceding 250 colonies) were not used. Plates with crowded colonies distribution (acceding 250 colonies) were not used. A set of control plates and a UV treated plate is shown in Figure S1.

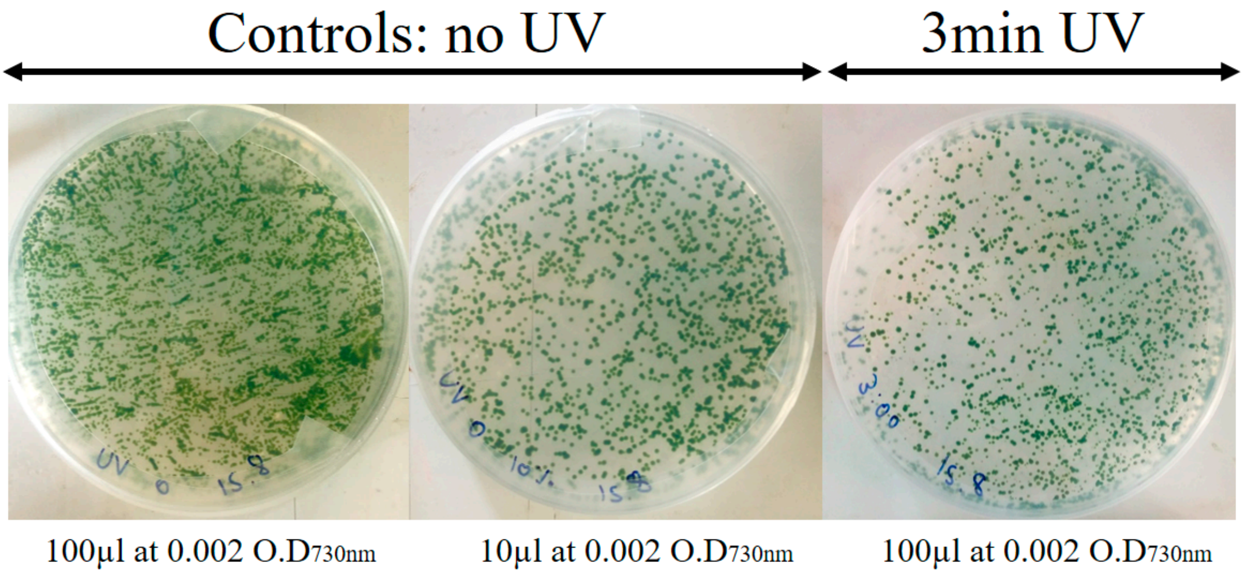

Figure S1. UV mutagenesis on *C. reinhardtii* cells. On the left two control plates, not subjected to UV treatment, one inoculated with 100µl of diluted liquid cell culture and the other with 10µl of the same culture (representing 10% of the cells of the first). On the right, a plate inoculated by 100µl of the same culture and placed at UV irradiation for 3 minutes, by which 90% of the cells died. The remaining 10% were taken to be screened by negative selections.

Negative selections for temperature sensitive photo-autotrophy

Single cell originated colonies of the randomly mutagenized strains were picked (with a simple toothpick), in a sterile environment (biological hood) and placed one by one in different wells of the 96WP and stirred in 100 µl of filtered DDW. 20µl of each single colony suspension were placed to cultivate in a parallel manner on two TAP-agar plates (one was placed at 37˚C and the other at 25˚C) and one TAP+ agar plate (that was placed at 37˚C). The colonies were placed respectively on the different mediums so after a few days under continues illumination the growth patterns of the different colonies were analyzed. Mutant colonies that grew on TAP- at 25°C and on TAP+ at 37˚C but did not grow on TAP- at 37˚C were selected.

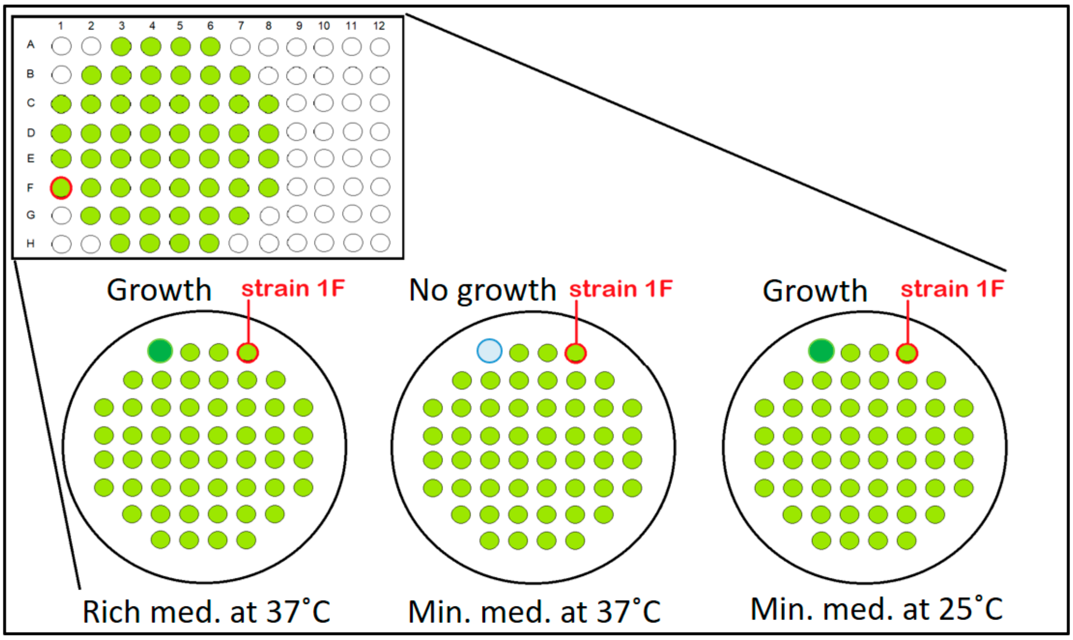

Figure S2. Negative selections for temperature sensitive photo-autotrophy: illustration of the parallel placement of mutated strains on 3 conditions: carbon rich medium at non permissive temperature of 37˚C (left), on a minimal medium at the non-permissive temperature (middle) and on a minimal medium at the permissive temperature of 25˚C (right). The parallel placement is shown by strain 1F taken from the bottom left position of the 96well and placed at the upper right position in all of the screening plates. The desired phenotype of temperature sensitive photo-autotrophy is illustrated by the strain at the upper left position showing growth on minimal medium only at permissive temperature conditions, while surviving at the non- permissive temperature on rich carbon supplied medium.

Statistical analysis for figures 1 and 2

Graphs were produced using Excel[50]. Statistical analysis of the resulted rates was performed using Graph Pad[51]. Paired t-test was performed for "37°C vs 25°C" and "recovery vs 25°C" for each strain (tables S1a and S2a). Unpaired t-test was performed for "TSP4 vs WT" and "TSP9 vs WT" for each temperature (tables S1b and S2b).

Column B vs. Column A	WT at 37°C vs. WT at 25°C	WT at rec vs. WT at 25°C	TSP4 at 37°C vs. TSP4 at 25°C	TSP4 at rec vs. TSP4 at 25°C	TSP9 at 37°C vs. TSP9 at 25°C	TSP9 at rec vs. TSP9 at 25°C
**Paired t test**						
**P value**	**0.5432**	**0.5841**	**0.0042**	**0.1652**	**0.0001>**	**0.0084**
**P value summary**	**ns**	**ns**	**	**ns**	****	**
**Significantly different (P < 0.05)?**	**No**	**No**	**Yes**	**No**	**Yes**	**Yes**
**One- or two-tailed P value?**	Two-tailed	Two-tailed	Two-tailed	Two-tailed	Two-tailed	Two-tailed
**t, df**	t=0.6636, df=4	t=0.6114, df=3	t=5.873, df=4	t=1.827, df=3	t=14.07, df=5	t=4.833, df=4
**Number of pairs**	5	4	5	4	6	5
**How big is the difference?**						
**Mean of differences (B - A)**	-16.78	-12.62	-157.7	-50.08	-108.4	-13.35
**SD of differences**	56.55	41.27	60.05	54.83	18.87	6.176
**SEM of differences**	25.29	20.64	26.85	27.42	7.704	2.762
**95% confidence interval**	-87.00 to 53.43	-78.29 to 53.05	-232.3 to -83.15	-137.3 to 37.16	-128.2 to -88.59	-21.02 to -5.680
**R squared (partial eta squared)**	0.09917	0.1108	0.8961	0.5266	0.9754	0.8538
**How effective was the pairing?**						
**Correlation coefficient (r)**	0.1748	0.7362	0.3743-	0.6516	0.4319-	0.9439
**P value (one tailed)**	0.3893	0.1319	0.2674	0.1742	0.1962	0.0079
**P value summary**	ns	ns	ns	ns	ns	**
**Was the pairing significantly effective?**	No	No	No	No	No	Yes

Table S1a. Paired t-test statistical analysis of O_2 production rates shown in Figure 1. Each pairing is listed on top. The analysis parameters and results are given for each case.

Column B vs. Column A	TSP4 at 25°C vs. WT at 25°C	TSP9 at 25°C vs. WT at 25°C	TSP4 at 37°C vs. WT at 37°C	TSP9 at 37°C vs. WT at 37°C
**Unpaired t test**				
**P value**	**0.9442**	**0.0057**	**0.0001>**	**0.0001>**
**P value summary**	**ns**	**	****	****
**Significantly different (P < 0.05)?**	**No**	**Yes**	**Yes**	**Yes**
**One- or two-tailed P value?**	Two-tailed	Two-tailed	Two-tailed	Two-tailed
**t, df**	t=0.07227, df=8	t=3.603, df=9	t=9.077, df=10	t=12.82, df=12
**How big is the difference?**				
**Mean of column A**	186.6	186.6	171.7	171.7
**Mean of column D**	184.2	113.6	30.29	6.172
**Difference between means (D - A) ± SEM**	-2.386 ± 33.01	-73.01 ± 20.26	-141.4 ± 15.58	-165.5 ± 12.91
**95% confidence interval**	-78.52 to 73.74	-118.8 to -27.17	-176.1 to -106.7	-193.7 to -137.4
**R squared (eta squared)**	0.000653	0.5906	0.8918	0.932
**F test to compare variances**				
**F, DFn, Dfd**	1.430, 4, 4	10.15, 4, 5	9.613, 5, 5	35.62, 5, 7
**P value**	0.7376	0.0257	0.0267	0.0002
**P value summary**	ns	*	*	***
**Significantly different (P < 0.05)?**	No	Yes	Yes	Yes
**Data analyzed**				
**Sample size, column A**	5	5	6	6
**Sample size, column D**	5	6	6	8

Table S1b. Unpaired t-test statistical analysis of O_2 production rates shown in Figure 1. Each strains rates were compared to the WT rates of the same temperature as listed on top. The analysis parameters and results are given for each case.

Column B	WT at 37°C	WT at rec	TSP4 at 37°C	TSP4 at rec	TSP9 at 37°C	TSP9 at rec
vs.	vs.	vs.	vs.	vs.	vs.	vs.
Column A	WT at 25°C	WT at 25°C	TSP4 at 25°C	TSP4 at 25°C	TSP9 at 25°C	TSP9 at 25°C
Paired t test						
P value	0.0671	0.7924	0.0001>	0.1763	0.0039	0.3258
P value summary	ns	ns	****	ns	**	ns
Significantly different (P < 0.05)?	No	No	Yes	No	Yes	No
One- or two-tailed P value?	Two-tailed	Two-tailed	Two-tailed	Two-tailed	Two-tailed	Two-tailed
t, df	t=2.232, df=6	t=0.2752, df=6	t=51.57, df=6	t=1.532, df=6	t=5.062, df=5	t=1.089, df=5
Number of pairs	7	7	7	7	6	6
How big is the difference?						
Mean of differences (B - A)	-0.04116	-0.003772	-0.5339	0.03254	-0.2535	-0.01112
SD of differences	0.0488	0.03625	0.02739	0.05618	0.1227	0.025
SEM of differences	0.01844	0.0137	0.01035	0.02123	0.05007	0.01021
95% confidence interval	-0.08629 to 0.003969	-0.03730 to 0.02976	-0.5592 to -0.5085	-0.01942 to 0.08449	-0.3822 to -0.1247	-0.03735 to 0.01512
R squared (partial eta squared)	0.4536	0.01247	0.9977	0.2813	0.8367	0.1917
How effective was the pairing?						
Correlation coefficient (r)	0.3813	0.5178	0.9396	0.8288	0.2152	0.7895
P value (one tailed)	0.1994	0.1169	0.0008	0.0106	0.3411	0.0309
P value summary	ns	ns	***	*	ns	*
Was the pairing significantly effective?	No	No	Yes	Yes	No	Yes

Table S2a. statistical analysis of maximum quantum efficiency of PSII (Max qe) shown in Figure 2. Each pairing is listed on top. The analysis parameters and results are given for each case.

Column B	TSP4 at 25°C	TSP9 at 25°C	TSP4 at 37°C	TSP9 at 37°C
vs.	vs.	vs.	vs.	vs.
Column A	WT at 25°C	WT at 25°C	WT at 37°C	WT at 37°C
Unpaired t test				
P value	0.992	0.2697	0.0001>	0.0032
P value summary	ns	ns	****	**
Significantly different (P < 0.05)?	No	No	Yes	Yes
One- or two-tailed P value?	Two-tailed	Two-tailed	Two-tailed	Two-tailed
t, df	t=0.01024, df=12	t=1.162, df=11	t=14.03, df=12	t=3.746, df=11
How big is the difference?				
Mean of column A	0.6685	0.6685	0.6273	0.6273
Mean of column D	0.6681	0.6899	0.1343	0.4364
Difference between means (D - A) ± SEM	-0.0003275 ± 0.03199	0.02141 ± 0.01842	-0.4930 ± 0.03515	-0.1909 ± 0.05095
95% confidence interval	-0.07004 to 0.06938	-0.01913 to 0.06195	-0.5696 to -0.4164	-0.3030 to -0.07874
R squared (eta squared)	8.73E-06	0.1094	0.9425	0.5606
F test to compare variances				
F, DFn, Dfd	3.574, 6, 6	2.948, 6, 5	5 2.852, 6, 6	7.021, 5, 6
P value	0.1464	0.2557	0.2278	0.0343
P value summary	ns	ns	ns	*
Significantly different (P < 0.05)?	No	No	No	Yes
Data analyzed				
Sample size, column A	7	7	7	7
Sample size, column D	7	6	7	6

Table S2b. Unpaired t-test statistical analysis of maximum quantum efficiency of PSII (Max qe) shown in Figure 2. Each strains rates were compared to the WT rates of the same temperature as listed on top. The analysis parameters and results are given for each case.

Growth rates and conditions for TSP mutants

The following figures show the growth rates of the WT, TSP4 and TSP9 strains under standard growth conditions

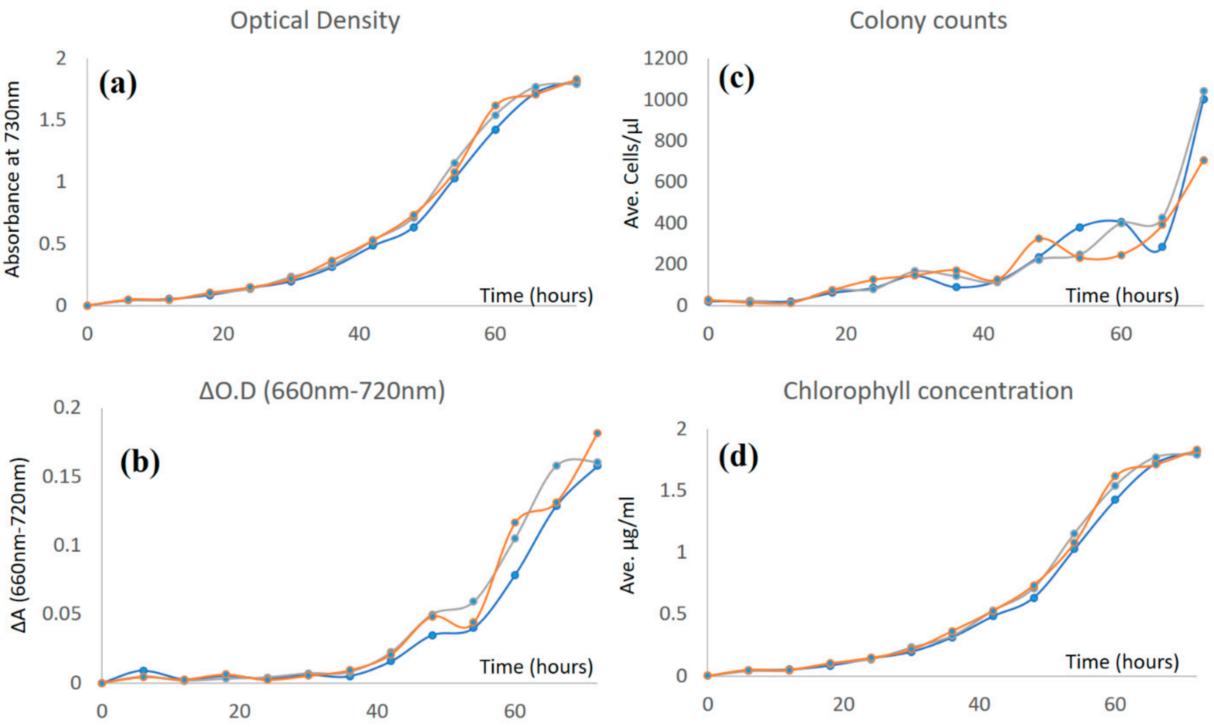

Figure S3a. Growth rates for WT, TSP4 and TSP9 at 25°C over time: seen by optical density (A), ΔA calculated as absorbance at 660nm-720nm (B), colony counts (C) and chlorophyll concentration (D). In all graphs the WT rate is shown in blue, TSP4 in grey and TSP9 in orange. Growth of Cells on TAP+ liquid cultures in the light was measured for 3 days. Cell density was determined every 6 hours.

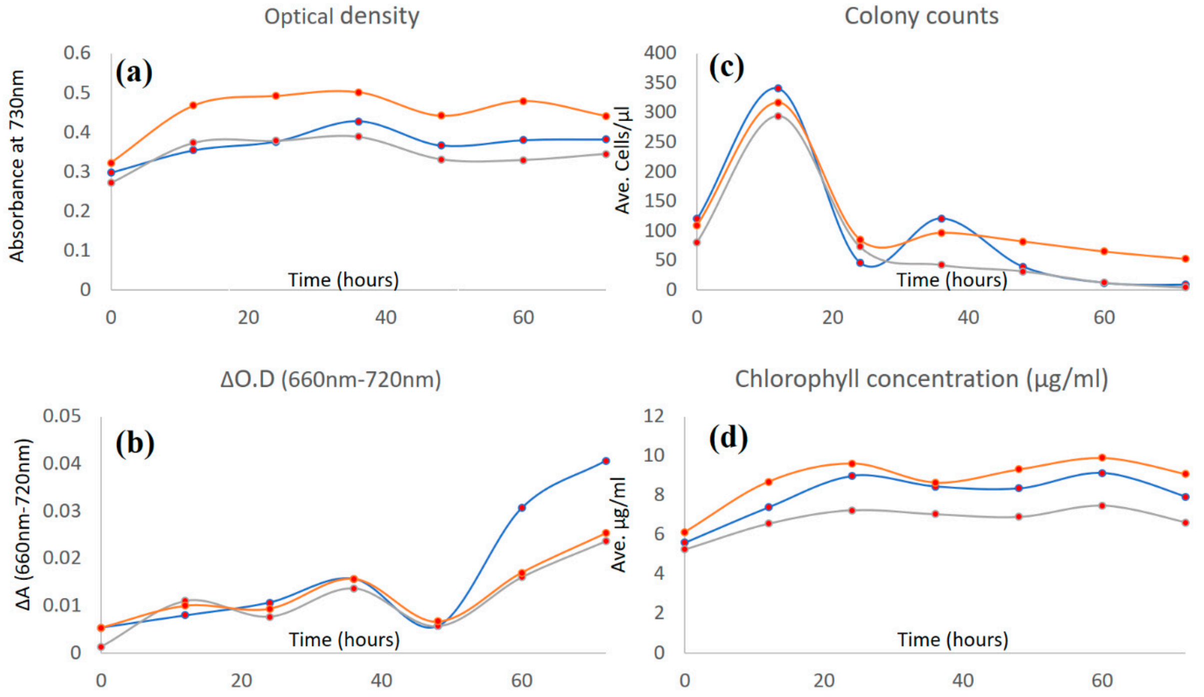

Figure S3b. Growth rates for WT, TSP4 and TSP9 at 37˚C over time: seen by optical density (A), ΔA calculated as absorbance at 660nm-720nm (B), colony counts (C) and chlorophyll concentration (D). In all graphs the

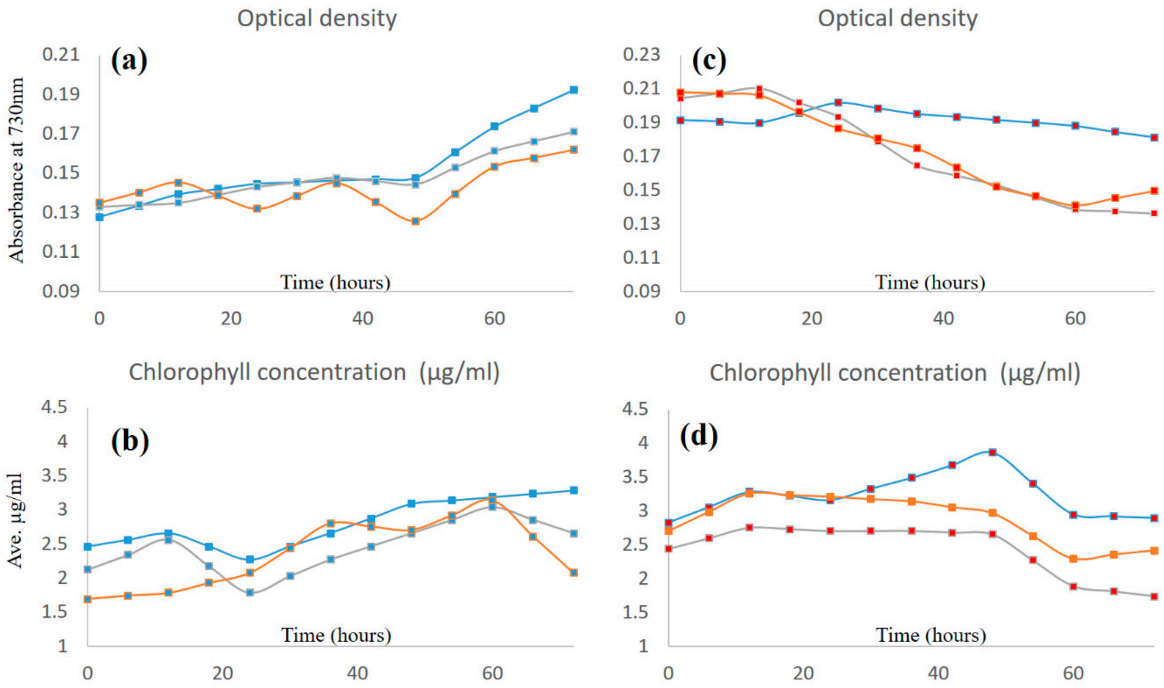

WT rate is shown in blue, TSP4 in grey and TSP9 in orange. Growth of Cells on TAP+ liquid cultures in the light was measured for 3 days. Cell density was determined every 12 hours.

Figure S3c. Photoautotrophic growth rates for WT, TSP4 and TSP9 at 25 ˚C (a and b) and at 37˚C)c and d) over time: seen by optical density (a and c) and chlorophyll concentration (b and d). In all graphs the WT rate is shown in blue, TSP4 in grey and TSP9 in orange. Growth of Cells on TAP- (no carbon in the medium) liquid cultures in the light was measured for 3 days. Cell density was determined every 12 hours.

Western blot analysis on isolated thylakoid membranes

Wild type of *C. reinhardtii* and the 6th generation (F6) of TSP9 mutant were grown in TAP+ medium at 25˚C, adapted to 37˚C for 14h and subsequently recovered to 25˚C for 24h. Thylakoids were isolated from each of the three conditions, lysed by SDS dissociation buffer and run on 17 % SDS-PAGE, transferred by a semi-dry transfer to cellulose membranes and subjected to immunoblotting. The amount loaded corresponded to chlorophyll content of 1.0 µg.

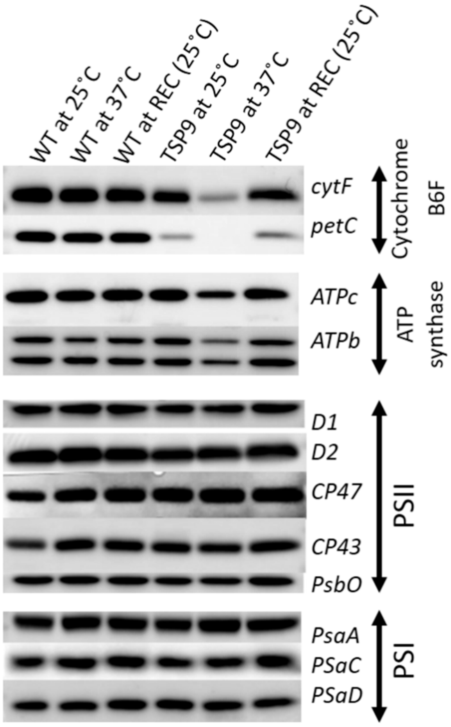

Figure S4. Immunoblots on isolated thylakoid membranes of WT and TSP9 grown at 3 temperature conditions:Antibodies against 12 subunits of four photosynthetic complexes: PSI, PSII, b6f complex and ATPase are lined on the right and on the left of them, in small letters, the various subunits are indicated. Expression levels of each complex of the mutant grown for 14 hours in 3 temperatures (indicated on top): 25˚C; 37˚C; grown at 37˚C and then grown for recovery at 25˚C, are shown. For each isolation process of a mutant strain a WT strain was used as control. Each of the different complexes was represented by a few subunits (3 for PSI, 5 for PSII, 2 for Cytochrome b6f and 2 for ATPase). Therefore, proteins present in the isolated membranes taken from cells grown at 3 temperature conditions are hereby compered.

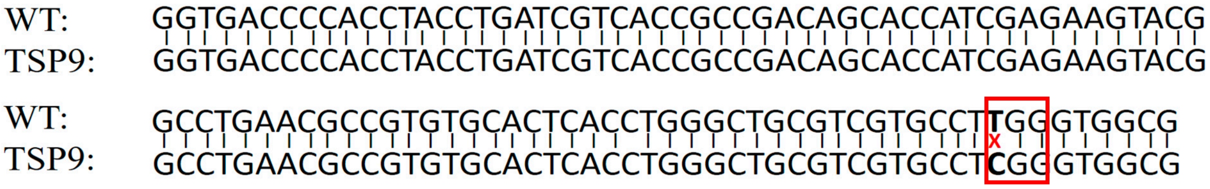

Figure S5. alignment of the TSP9 mutant sequence (bottom line) against the original gene [33] recorded for petC *C. Reinhardtii*.

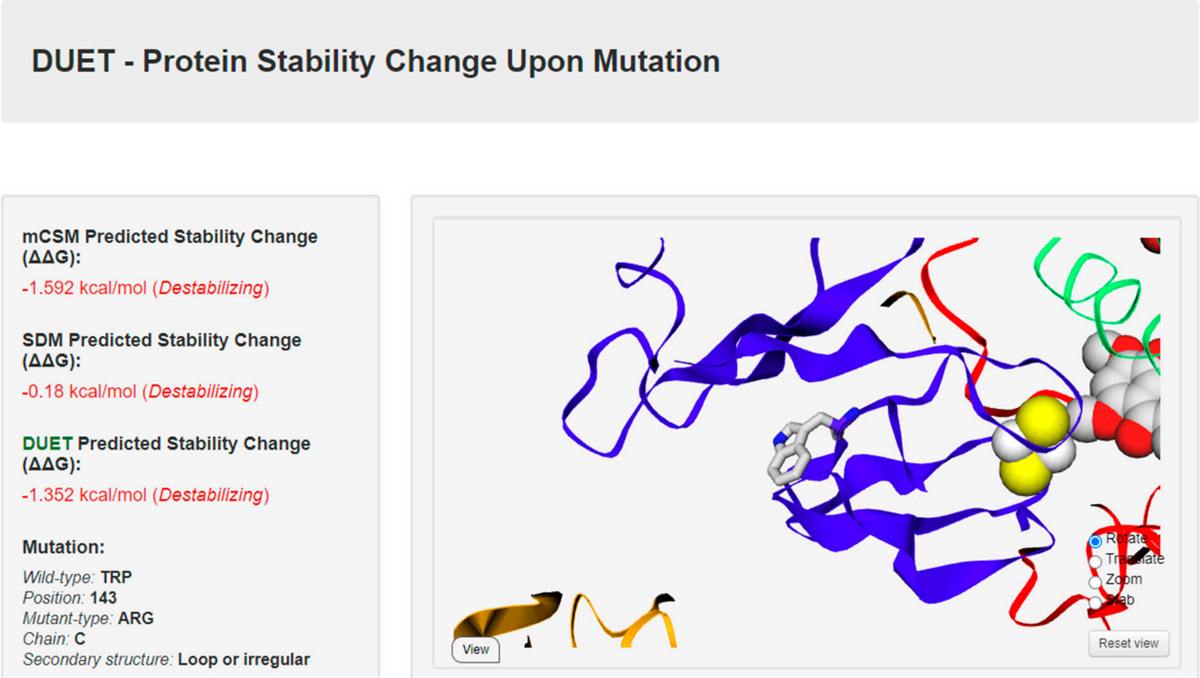

Figure S6a. Results of DUET analysis for protein stability predictions. The mutation site is shown in the middle of the frame and the resulted stability predictions are on the left. The change of the tryptophan to arginine according to this analysis should highly effect the protein stability.

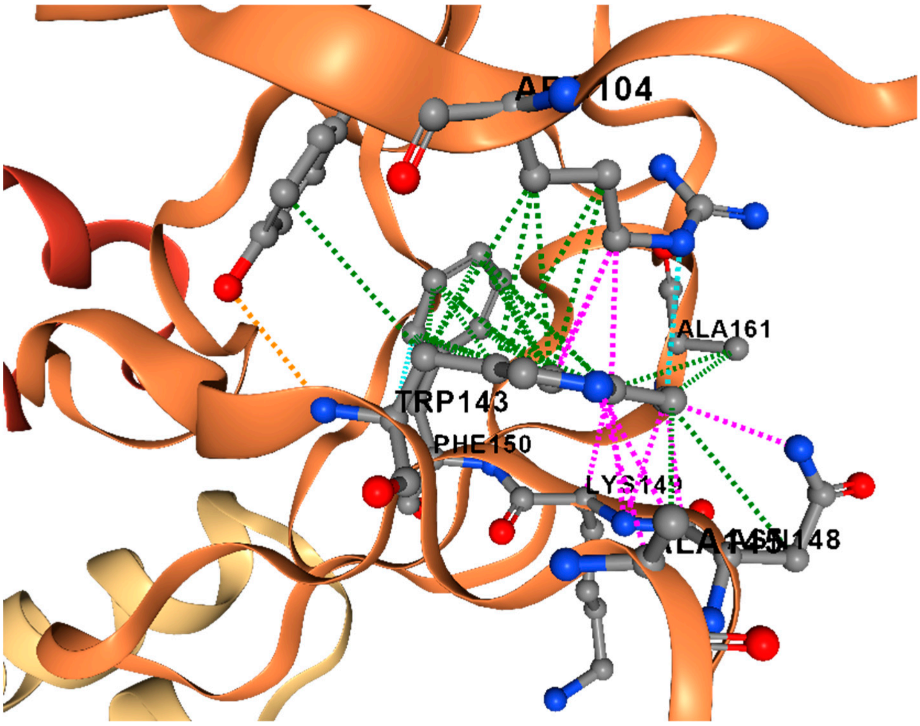

Figure S6b. Results of Dynamute2 analysis for protein stability predictions. The mutation site is shown in the middle of the frame and the interactions around it are marked by color coded dashed lines; Pink for clash interactions, green for hydrophobic interactions, orange for polar interactions, cyan for VDW interactions. The change of the

tryptophan to arginine according to this analysis should highly effect the protein stability.

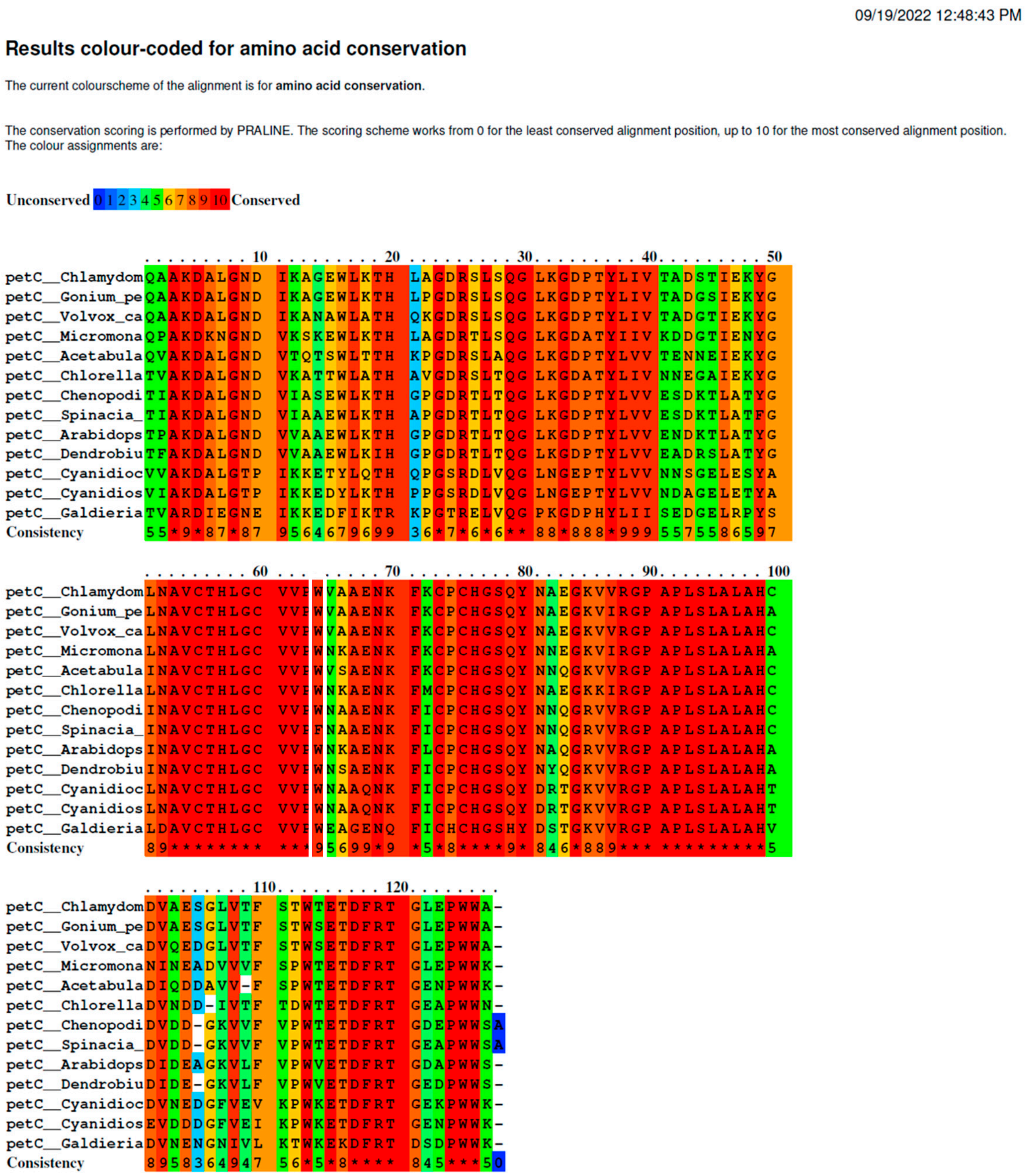

Figure S7. Full msa analysis by PRALINE (link to server in: https://www.ibi.vu.nl/) for petC gene of the rieske protein: the first sequence from the top is the sequence known for *C. reinhardtii*, followed by 12 other sequences for other organisms. In this alignment the tryptophan (W) in position 64, outlined in white, is the amino acid mutated in TSP9 and replaced to arginine (R). Here the position conservation score is 9/10, as it is coloured in light red. The only case (in spinach) where this position is occupied by a different amino acid, it is replaced by phenylalanine (F).

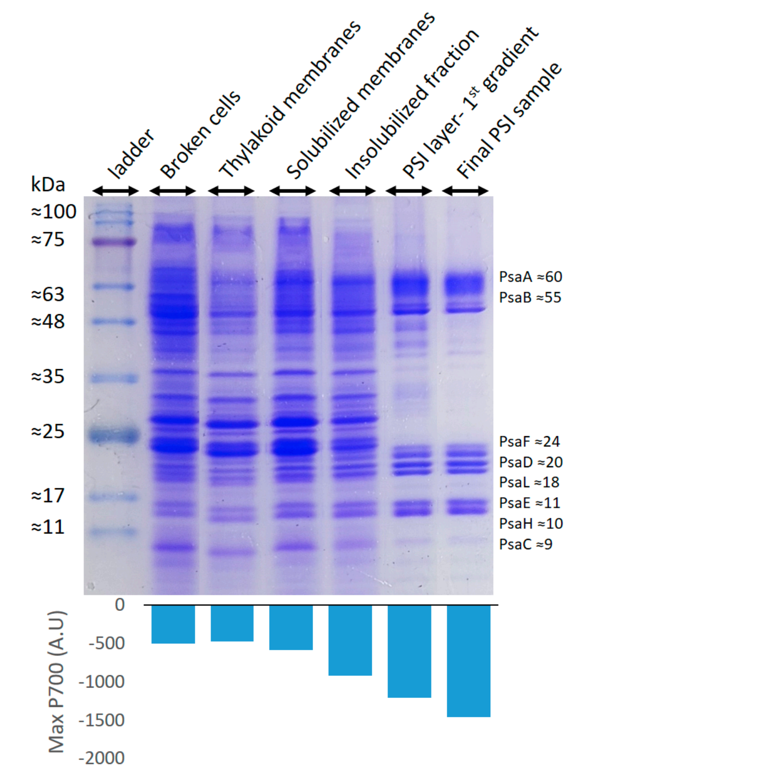

Figure S8. Gel staining of PSI purification steps and the corresponding P700 signal from TSP9 mutant grown at the non-permissive temperatures. 1μg of chlorophylls was loaded into each well of SDS gel and the general protein levels of each sample is shown by blue staining. A ladder is shown on the left, above the indicated size in kilo-Dalton (kDa) , possible subunits and their molecular size is given on the right. Each step is listed on top. The P700 max signal was collected by JTS-10

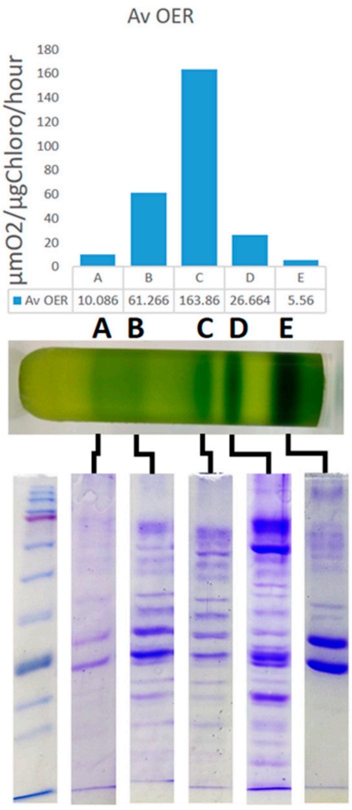

for 5µg of chlorophylls and given on the bottom in A.U units (Arbitrary Units).

Figure S9. The distribution of green bands in the 1st gradient tubes with the corresponding OER results and SDS-dissolved samples for general proteins shown by staining: each layer of the 20-50% sucrose was collected (marked A-E), the chlorophyll content was measured and balanced to the same levels. To allow PSII activity in the dissolved membranes, 5µg samples of chlorophylls from each layer were suspended for 1min in the dark with 350mM of 2,6 Dichloro-1,4-benzoquinone. Activity levels were measured by OERs using oxygraph plus (as before). Samples were dissolved by SDS and 1µg of chlorophylls were run on SDS-PAGE for general protein staining shown on the bottom of the figure.

Figure S10. Cryo-EM data processing workflow of TSP9-PSI-8LHC. (A) A zoomed-in view on a TSP9-PSI micrograph. Scale bar size is 200 Å. (B) Representing class averages after 2D classification. (C) chosen 3D classes, particles number and percentage out of all chosen particles are indicated on the bottom (D) color coded local resolution of the complete TSP9-PSI-8LHC and scale (under the model).

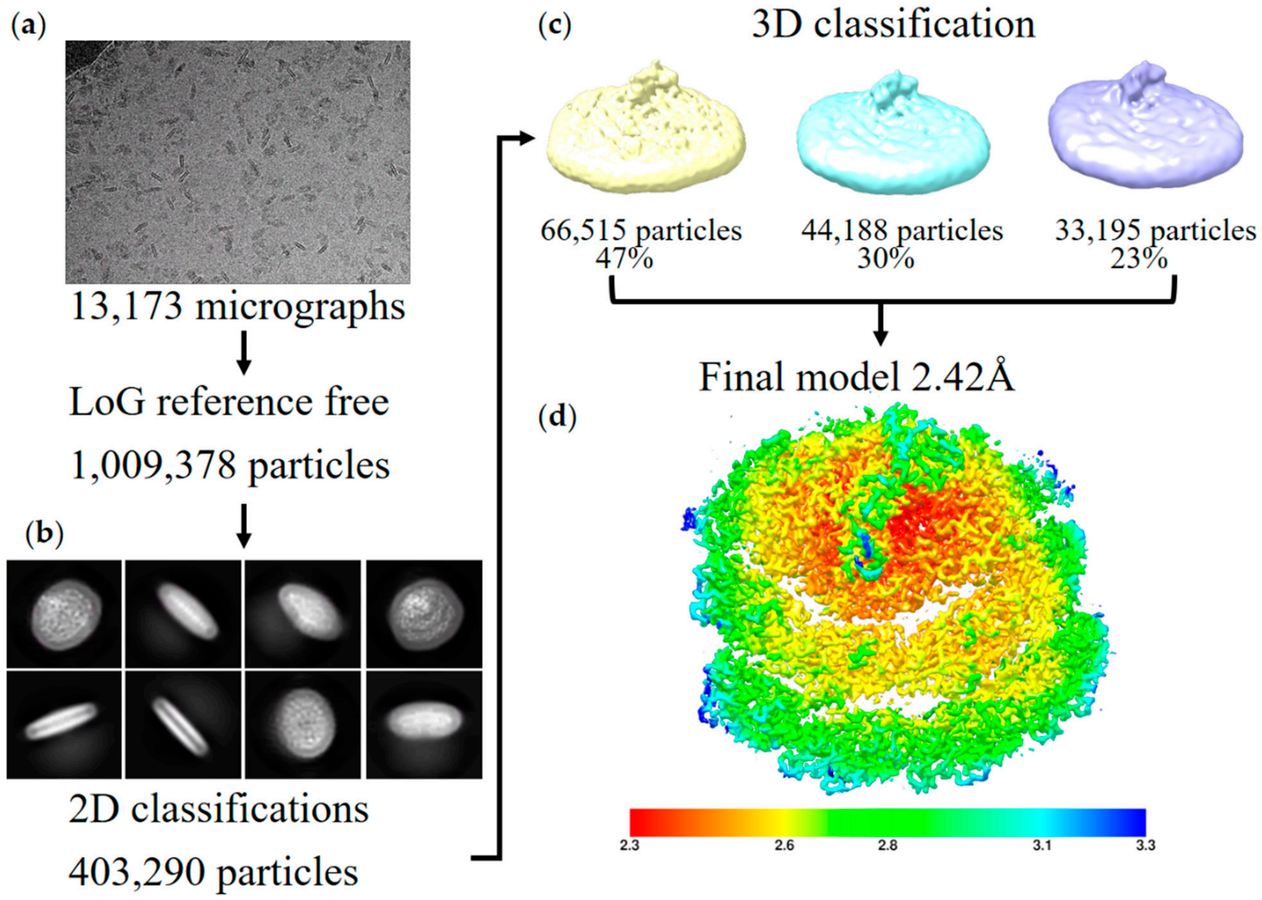

Supplement: Supplementary file 1 [file biomolecules-13-00537-s001.zip › Supplementary Materials_22022023.pdf]
